# Infants < 90 days of age with late-onset sepsis display disturbances of the microbiome-immunity interplay

**DOI:** 10.1007/s15010-024-02396-6

**Published:** 2024-11-14

**Authors:** Simon Graspeuntner, Mariia Lupatsii, Vera van Zandbergen, Marie-Theres Dammann, Julia Pagel, Duc Ninh Nguyen, Alexander Humberg, Wolfgang Göpel, Egbert Herting, Jan Rupp, Christoph Härtel, Ingmar Fortmann

**Affiliations:** 1 Department of Infectious Diseases and Microbiology, University of Lübeck, Lübeck, Germany; 2 German Center for Infection Research (DZIF), Partner Site Hamburg-Lübeck-Borstel-Riems, Lübeck, Germany; 3 Department of Pediatrics, University of Lübeck, Ratzeburger Allee 160, 23538 Lübeck, Germany; 4 Department of Pediatrics, University Medical Center Hamburg-Eppendorf, Hamburg, Germany; 5 Section for Comparative Pediatrics and Nutrition, Department of Veterinary and Animal Sciences, University of Copenhagen, Copenhagen, Denmark; 6 Department of Pediatrics, University of Münster, Münster, Germany; 7 Department of Pediatrics, University Hospital of Würzburg, Würzburg, Germany

**Keywords:** Neonatal immunity, Microbiome, Sepsis, Infants < 90 days, Bifidobacteria

## Abstract

**Objective:**

We hypothesized that previously healthy infants < 90 days of age with late-onset sepsis (LOS) have disturbances of the gut microbiome with yet undefined specific immunological patterns.

**Methods:**

We performed a prospective single-center convenience sample study between January 2019 and July 2021 in a case-control design. Routine diagnostics included conventional cultures (blood, cerebrospinal fluid, urine), PCRs and inflammatory markers in infants aged < 90 days with clinical LOS. We additionally analyzed blood lymphocyte subsets including CD4 + CD25 + forkhead box protein (FoxP3)^+^ Tregs and performed 16 S rRNA sequencing of stool samples, both compared to age-matched healthy controls. Results were adjusted for potential confounders that may influence microbial composition.

**Results:**

51 infants with fever and clinical LOS were enrolled. Bacterial sepsis was diagnosed in *n* = 24 (47.1%) and viral infection in *n* = 13 (25.5%) infants, whereas in 14 (27.3%) infants the cause of fever remained undetermined. When compared to healthy controls, the gut microbiome of LOS infants at disease onset was characterized by a shift in community composition, specifically, decreased abundance of *B. longum* and an increase of *Bacteroidia* spp. Intriguingly, the abundance of *B. longum* negatively correlated with the frequency of blood CD4-positive cells in healthy controls but not in infants with LOS. At one year of age, we observed microbiome differences in infants with history of LOS when compared to healthy controls, such as an increased gut microbial diversity.

**Conclusion:**

Our data suggest potential signatures of the microbiome-immunity interplay in infants with LOS, which should be investigated further as possible targets for prevention.

**Supplementary Information:**

The online version contains supplementary material available at 10.1007/s15010-024-02396-6.

## Introduction

Neonates under the age of 90 days are at an increased risk for sepsis, a frequent cause of morbidity and mortality [1]. In febrile infants < 90 days of age 2–15% have invasive bacterial infection (IBI) with urosepsis being the most common focus [2]. Diagnostic approaches such as biomarkers and cultures, which are conducted at hospital admission, are non-specific and cannot adequately guide the decision whether or not to initiate antibiotic treatment at the time of first contact [3]. Neonates and young infants are, therefore, subject to a high exposure rate to empirical antibiotic therapy [4, 5]. While the risk factors for healthcare-associated late-onset sepsis (LOS), particularly in preterm infants, have been extensively studied [6], there are only few data on risk factors and clinical manifestations of community-acquired LOS. These include prematurity [7], maternal colonization with group B streptococci [7], urinary tract malformations [8], underlying disease, age at admission < 28 days [9] and immune deficiencies. From studies with preterm infants it is known that certain compositional microbiome and metabolome characteristics play a central role for sepsis risk [10, 11]. Gut dysbiosis, specifically fewer bacterial species, less diversity and increased proportions of potential pathogens result in an altered barrier and immune function and is associated with LOS [11]. These aspects have not yet been investigated for previously healthy term infants with community-acquired LOS. However, there is convincing evidence that the microbiome, and its disturbance (often referred to as “dysbiosis”), is involved in the development of sepsis early in life [12, 13]. For example, the lack of *Bifidobacterium* spp. and reduced availability of human milk oligosaccharides (HMOs) are dysbiosis markers being associated with sepsis and systemic inflammation [14]. Approaches to resurrect the disturbed early microbiome by supplementation with *Bifidobacterium *spp.-containing probiotics have been successfully established for preterm infants and may prevent inflammatory disorders such as necrotizing enterocolitis [9]. While animal models show sepsis-preventive probiotic effects [15], the effect of probiotic supplementation to prevent LOS in human young infants is uncertain and may depend on human milk feeding [16]. Of note, the interdependence between the development of the immune system and the microbiome is highly dynamic in the first months of life [17]. It is yet unknown whether specific microbiome signatures, i.e. reduced microbial diversity and lack of *B. longum*, are associated with immunological features contributing to LOS development in previously healthy term infants < 90 days of age with LOS.

Historically, neonatal immune functions were considered immature and less functional. Current concepts, however, describe balanced, adaptive processes of defense against invasive pathogens and tolerance towards commensal colonization, which is particularly mediated by CD4 + and CD8 + cells [18]. Regulatory T cells (Tregs) regulate the ontogenetic control of immune activation and feto-maternal tolerance, limiting the immune response against self- and non-self-antigens [19]. However, their immunosuppressive capacity can also contribute to an increased susceptibility to LOS [20]. The roles of B cells and NK cells in neonatal sepsis are not fully understood. Reduced NK cell levels at birth are associated with an increased risk of LOS. The unique immune responses of newborns play a crucial role in transitioning from the womb to the antigen-rich outside world, with age-dependent susceptibility to infection being a significant concern. For young infants with LOS, we have recently described specific immunological features, including a lack of regulatory T cells, supporting the concept of immaturity and failure of immune tolerance development [2]. Since Tregs contribute to the regulation of permissive physiological colonization of young infants [21–23], we herein speculate that a lack of Tregs may represent a feature of dysregulated immune-microbiome interplay in infants with LOS.

## Materials and methods

### Study design and methods

We performed a prospective single-center case-control study (Fever Without Source project; FWS project) using a convenience sample of infants admitted from home for suspected LOS and without a history of previous infections in the Department of Pediatrics at the University Hospital in Lübeck. Immunological data from a subset of infants enrolled in the FWS project were previously published in a manuscript in *Frontiers in Immunology* [2]. Although there is an overlap with the cohort presented in this study (44 out of 51 patients; 86.3%), it should be mentioned that the current study cohort represents an extension of the previously reported group. For the current analysis, we recruited infants from January 1st, 2019, until July 1st, 2021, including only infants for whom full datasets of immunological as well as microbiome data were available.

### Participants

Infants aged < 90 days and > 72 h with suspected LOS, undergoing sepsis workup, and empirical antibiotic treatment met the inclusion criteria within the FWS project. A subgroup of these infants was observed until the age of one year. As age-matched controls we included infants without infectious symptoms that were admitted for non-infectious reasons such as elective surgery or conditions requiring clinical monitoring in the hospital. For the follow-up at 1 year of age, a different set of healthy control infants without a history of sepsis were recruited from a pediatric practice.

### Data collection

Following parental (or legal representatives’) provision of written informed consent, the doctors admitting the patients registered the infants with suspected LOS and the corresponding controls. The attending physician was in responsible for documenting the patient history and demographic details, clinical symptoms and clinical findings on a written case report form. Relevant details of the clinical course were completed by the study physician who as well monitored all documented data against the original patient files and transferred the pseudonymized data into a prespecified Microsoft Excel database (Microsoft Office 2010, Versions 14.0).

### Follow-Up and sample collection

The follow-up examination at one year of age consisted of a parent report on development, morbidities (e.g. infections, antibiotic exposure), social environment and nutrition during the first year of life, a physical examination and acquisition of a stool sample for microbiome diagnostics.

### Diagnostic procedures

To categorize our cohort into subgroups based on the underlying cause of infectious symptoms, we expanded our standard sepsis diagnostics, which included inflammation markers and cultures of blood, urine, and cerebrospinal fluid. Stool samples of infants presenting symptoms of gastroenteritis were screened for rotavirus, adenovirus and norovirus using antigen tests (Ridascreen, r-Biopharm, Darmstadt, Germany). Further, detailed microbial testing included an in-house multiplex PCR analysis of nasopharyngeal aspirates (NPA) and an enterovirus PCR at the local Institute for Microbiology using stool samples. The NPA multiplex assay (RP2Plus Biofire^®^ respiratory 2.1 plus panel) covered a range of pathogens including adenovirus, human rhino-/enterovirus, respiratory syncytial virus (RSV), influenza virus A and B, parainfluenza virus 1–4, metapneumovirus, coronavirus (with subtypes HKU1, NL63, 229E, OC43), MERS CoV, *Bordetella pertussis*, *B. parapertussis*, *Chlamydia pneumoniae*, and *Mycoplasma pneumoniae*.

### Ethics

Written informed consent was obtained from the parents or legal guardians of the infants participating in the study before enrolment of their children. Approval for the study components was granted by the local ethics committee for research involving human subjects at the University of Lübeck. The reference number for the FWS project is 20–228. The trial protocol adhered to the Guidelines for Good Clinical Practice (GCP) and the Declaration of Helsinki. Blood samples were collected solely during medically indicated blood draws. The additional blood volume per sampling was less than 1% of the total blood volume, which is in accordance with the guidelines of the European Medical Agency (EMA) for investigating medicinal products in term and preterm infants as stipulated by the Committee for Medicinal Products for Human Use and Pediatric Committee (PDCO, 2009).

### Definitions

**Late-onset sepsis** (LOS) was defined as sepsis occurring after the first 72 h of life. **Culture-confirmed sepsis** and **invasive bacterial infection** (IBI) were defined as clinical sepsis with proof of causative agent in cultures of blood, urine or cerebrospinal fluid. **Viral infection** was defined as proof of causative virus via multiplex PCR from nasopharyngeal aspirate, enterovirus PCR from stool samples, RSV rapid test, influenza rapid test or PCR test for adenovirus, norovirus and rotavirus. **Fever** was defined as central (rectal) body temperature of > 38.0 °C. **Gestational age** was calculated from the best obstetric estimate based on early prenatal ultrasound and obstetric examination.

### Microbiome samples

After hospital admission and parental consent, we collected the first fecal sample that was available for microbiome analyses in order to minimize the impact of empirical antibiotic treatment. All infants received a triple combination of ampicillin, gentamicin and cefotaxime as recommended by the current German guideline [24]. Stool samples were collected within a median of 7 h (IQR 3–9.5 h). Control samples were collected from afebrile infants, who were admitted to hospital for non-infectious reasons (i.e., for elective surgery) and had not been exposed to antibiotic treatment. The stool samples from infants with sepsis and controls were prepared using the same methods: the fresh stool samples were placed in sterile DNAase-/RNAase-free Eppendorf tubes, immediately stored in a -20 °C freezer before being transferred to a -80 °C freezer within 2 days. Fecal samples of infants with 12 months follow-up were collected by the infants’ parents at home and were likewise immediately stored in a -20 °C freezer. Material for sampling along with instructions on how to correctly collect the sample was sent to the parents in advance. Parents were asked to collect the sample no earlier than the day before follow-up. For transportation of the sample to the hospital parents received a cool bag and cool packs to avoid thawing of the sample. At the hospital, samples were immediately frozen at -80 °C. This strategy was used to maintain sample quality during transport to the clinic and aimed at preventing any thawing until the samples were stored at -80 °C in the clinic. For families living close to the clinic, a non-electric cool box was used for transport. For families living further away, we collected the samples from their homes and used an electric (-60 °C) freezer for transport. This approach ensured that the samples remained frozen during transit.

To establish a control cohort for our 12-month follow-up, randomly chosen families were asked to participate in the FWS study in a pediatric practice in Lübeck (R.O.). In the case of consent, fecal samples were picked up at the family’s homes as described above.

### DNA isolation in fecal samples

After thawing the fecal samples, approximately 100-200 mg of stool samples were processed using DNeasy^®^ PowerSoil^®^ Pro DNA Isolation Kit (Qiagen GmbH, Hilden, Germany). Isolation was performed according to the manufacturer`s protocol. With each round of isolation, we ran a negative isolation control to control for reagents contamination. The isolated DNA was stored at − 20 °C.

### Amplification via polymerase chain reaction (PCR) and partial 16 S rRNA gene sequencing

16 S rRNA gene sequences of isolated DNA samples were amplified using linker and indices-containing primers targeting the 16 S rRNA gene’s V3/V4 hypervariable regions. The primer design is given elsewhere [25] and primer sequences are given in Supplementary Table 1. Polymerase chain reaction (PCR) quantification of amplicons and library preparation were performed as described in previous work by our group [11, 25, 26]. Sequencing was performed using MiSeq^®^ platform (Illumina^®^, San Diego, California, USA) and MiSeq^®^ reagent Kit V3 (600 cycles using PhiX library as a positive control). Negative isolation controls ensured that reagents were not contaminated. Samples that subsequent to PCR showed a definable amplicon were subject to data processing and analysis while isolation controls remained negative.

### Bioinformatics

Using mothur version 1.44.1, raw fastq files were processed [27]. Bioinformatic processing was performed following a previously established protocol [28]. Briefly, contigs were screened to not exceed the length of 500 bp and not contain ambiguous bases and were subsequently aligned against the SILVA reference data base [29]. Thereafter, sequences with more than 12 homopolymers were filtered out and chimeric sequences removed using VSEARCH [30]. Unique sequences were classified using the Green genes Data Base [31] applying a 97% assignment cutoff and rarified at 4300 reads per sample after removal of sequences classified as chloroplast, mitochondrial, archaeal and eukaryotic.

### Analyses of white blood cell counts, lymphocyte subsets and Tregs

For the current study, we only included blood samples that were taken at hospital admission from infants enrolled in the FWS project along with the medically indicated blood samples taken by the attending physician. In accordance with the usual standards for pre-analytical time windows in clinically oriented immunological laboratories, these EDTA whole blood samples were stored at room temperature for a maximum period of 24 h before they were processed either in the central laboratory of the University Hospital (lymphocyte subsets, blood counts) or in the research laboratory of the pediatric department (regulatory T cells). The samples were processed in the same manner across all subgroups and controls.

The methodology for determining regulatory T cells via flow cytometry in our immunological research laboratory has been described in detail in previous publications by our research group [2, 20, 32]. In general, CD3 + lymphocytes, CD4 + lymphocytes, CD3 + CD4 + lymphocytes, CD3 + CD4 + CD25 + lymphocytes, CD3 + CD4 + Foxp3 + lymphocytes and CD3 + CD4 + Foxp3 + CD25 + lymphocytes were analyzed. Before staining, cell viability tests were performed to control for dead cells and to ensure cell viability after 24 h (eBioscience, San Diego, CA, USA). Subsequently, 100 µl whole blood were stained with fluorochrome-labeled antibodies to characterize T cell populations using Surface CD3 (fluorescein isothiocyanate, FITC; eBioscience), CD4 (phycoerythrin, PE; Miltenyi Biotec, Bergisch Gladbach, Germany), and CD25 (brilliant violet, BV421; BioLegend, San Diego, CA, USA). After surface staining, we proceeded with fixation followed by permeabilization using the appropriate cell permeabilization and fixation reagents (FoxP3/Transcription Factor Staining Buffer Set; eBioscience, Thermo Fisher Scientific, Waltham, MA, USA). Then, intranuclear staining for FoxP3 (eFluor660; eBioscience) was performed according to the manufacturer protocols. The fixed and stained cells were directly stored at 4 °C in FACS staining buffer (eBioscience) until multicolor flow cytometric analysis, which was performed within 4 days. The fixed and stained cells were directly stored at 4 °C in FACS staining buffer (eBioscience) until multicolor flow cytometric analysis, which was performed within 4 days. Flow cytometry for determination of the cell counts and population percentages was carried out on a BD LSR II cytometer using FACS Diva software (BD Bioscience, San Jose, CA, USA) and FlowJo (Tree Star, Ashland, OR, USA; Version 10.7.0). These were identified by their position in the forward-/side-scatter plot (size/granularity) and co-expression of CD3, CD4, CD25 and FoxP3. Fluorescence minus one (FMO) controls were used to establish gating boundaries and assess background fluorochrome spread. Representative plots of our gating strategy for flow cytometry analysis of CD3+, CD4+, CD25+, forkhead box protein 3 (FoxP3+) regulatory T cells are shown in Supplementary Fig. 3.

Blood counts and further lymphocyte characterization were performed in our central laboratory of the University Hospital. A BD FACS Canto II system (BD Bioscience, San Jose, CA, USA) equipped with the BD FACS Canto Clinical software was used for further analysis of lymphocyte subsets (CD8+/CD19+). Analysis of lymphocyte subsets and their activation status in whole blood utilized Multitest 6-Color TBNK (T cells and B cells) kits and Multitest CD3/CD8/CD38/HLA-DR kits according to the manufacturer’s protocols. Representative plots and detailed information on the gating strategy used by our central laboratory at the university hospital are available in the BD Biosciences user manual. Weekly checks of all cytometer performances were performed using BD FACS 7-Color Setup Beads, with alternating use of BD Multi-Check Control and BD Multi-Check CD4 Low Control quality controls twice daily.

### Statistical analysis

The analyses of this study were conducted using the most recent version of SPSS software (Version 29.0; SPSS Inc., Munich, Germany) and R (version 4.0.1). For descriptive statistics, data were summarized as either percentages or medians with interquartile ranges. Differences between groups were analyzed using Pearson’s Chi-square test or Fisher’s exact test for categorical variables, while the Mann–Whitney U test was used for continuous variables. Statistical significance was set at a p-value of less than 0.05 for all tests. Further statistical analysis and graphical visualization were assembled via R (version 4.0.1) using the packages vegan [33] and labdsv [34]. Alpha diversity measurements were assessed using Shannon’s diversity index and by calculating the number of detected species in each of the samples. Differences in alpha diversity measures and relative abundance between groups were assessed using a non-parametric analysis of covariance (ANCOVA) [35] adjusting for potential confounding factors that varied between the control and the LOS groups. Specifically, the variables considered were delivery mode, age at hospital admission, age at follow-up, and human milk feeding at the time point of LOS onset. Additionally, for the 1-year analysis, we accounted for antibiotic exposure, duration of human milk feeding, and probiotic administration (see Table 1 for the distribution of variables).

**Table 1:** Clinical characteristics of study cohort

	Clinical LOS	Control group^#^	*p*
**Number** n	51	40	♦
**Gestational age** (weeks; median, IQR)	39.0 (37.5–40.3)	39.2 (38.0–40.1)	0.6
**Birth weight** (g; median, IQR)	3770 (3005–3740)	3530 (3201–3690)	0.4
**Gender**,** male** (%, n)	50.9 (*n* = 26)	60.0 (*n* = 25)	0.4
**Delivery mode**,** vaginal delivery** (%, n)	78.4 (*n* = 40)	62.5 (*n* = 25)	0.09
**Age at admission/sampling** (days; median, IQR)	46.0 (12.0–57.0)	38.0 (28.0–42.0)	0.1
**Antenatal antibiotic exposure** (%, n)	25.5 (*n* = 13)	20.0 (*n* = 8)	0.5
Previous antibiotic exposure* (%, n)	13.7 (*n* = 7)	♦	♦
**Human milk-fed** (%, n)	70.5 (*n* = 36)	82.5 (*n* = 33)	0.2
**Exclusively HM-fed** (%, n)	41.1 (*n* = 21)	67.5 (*n* = 27)	0.01
**Formula-fed** (%, n)	58.8 (*n* = 30)	32.5 (*n* = 13)	0.01
**Exclusively formula-fed** (%, n)	29.4 (*n* = 15)	17.5 (*n* = 7)	0.2
**Human milk and formula nutrition** (%, n)	29.4 (*n* = 15)	15.0 (*n* = 6)	0.1
**Antibiotic therapy for LOS** (%, n)	100 (*n* = 51)	♦	♦
**Duration of antibiotic therapy** (days; median, IQR)	7.2 (4.1–10.2)	♦	♦

IQR, interquartile range; HM, human milk; **#** p-value were derived. from Chi-Square test or for continuous variables Mann-Whitney U test

*Antibiotic exposure for suspected but unconfirmed early-onset sepsis

^♦ Exclusion criteria for controls or not applicable^

We adjusted the comparisons for relative abundances to the number of taxa tested by using the p-adjust-function in R with the method set to “fdr” (false-discovery rate adjustment) to control for type 1 error inflation. In addition, we performed analyses based on a hierarchical procedure: species level taxa were analyzed only if they were belonging to a genus which was significantly different between sepsis and control cases. Beta diversity was analyzed using principal coordinates analysis generated with Bray-Curtis dissimilarities. To assess the impact of LOS on data variance, constrained correspondence analysis was used using LOS status and potentially confounding variables (as described above) as constraints. Differences between groups were calculated via permutational multivariate analysis of variance using distance matrices. Identification of indicator species was performed by Linear Discriminant Analysis Effect Size (LEfSe) provided by the Galaxy Project Platform [36, 37]. To compute the correlation of immune cell markers with the abundance of the genus *Bifidobacterium* and the species B. longum, Pearson correlation coefficient was calculated with subsequent derivation of R² and p-values using psych package [38]. To further evaluate the correlation between bacterial abundance and immune cell marker counts in the LOS and control group separately, a multivariate linear model was computed fitting all immune cell measurements screened via the stats package.

## Results

### Study cohort

Between January 1st 2019 and July 31st 2021, *n* = 105 infants were screened as possible candidates for participation in the FWS project. *N* = 54 infants were excluded due to the following reasons: not approached (*n* = 19), denied consent (*n* = 21), known underlying immunological disease (*n* = 1), incomplete dataset (*n* = 13). Within the control group *n* = 40 out of 55 approached families were included in the study.

### Clinical characteristics of study cohort

In our study *n* = 51 infants < 90 days of age with fever and suspected LOS were included. The median gestational age was 39.0 (IQR 37.5–40.3) weeks, with a median birth weight of 3700 (IQR 3005–3740) grams (Table 1). The median age at hospital admission was 46.0 (IQR 12.0–57.0) days. 40 infants (78.4%) were born via spontaneous delivery, 26 (50.9%) were male and 7 infants (13.7%) had a history of postnatal antibiotic exposure for non-confirmed early-onset sepsis for a maximum of 3 days. The majority of infants were fed with human breast milk, 41% exclusively. All infants with suspected LOS were empirically treated with antibiotics at hospital admission for a median duration of 7.2 days. IBI was diagnosed in 24 out of 51 infants (47.1%) whereas 25.5% had proven viral infection (Table 2). In 14 infants (27.4%) no bacterial or viral pathogen was found and the cause of fever remained undetermined.

**Table 2:** Bacterial and viral causes of suspected LOS in infants < 90 days of age

	Invasive bacterial infection		Viral infection	
	*n* = 24 (47.1%)		*n* = 13 (25.5%)	
**Focus**	Urosepsis BC-positive Meningitis Endocarditis Pneumonia	*n* = 17 *n* = 7 *n* = 1 *n* = 1 *n* = 1	Respiratory infection Meningitis Pneumonia Gastroenteritis	*n* = 9 *n* = 4 *n* = 3 *n* = 1
**Pathogen**	*E. coli* *GBS* *Kl. oxytoca* *Kl. pneumoniae*	*n* = 17 *n* = 5 *n* = 1 *n* = 1	Enterovirus Rhinovirus Rotavirus RSV Corona NB Corona NL 63	*n* = 6 *n* = 3 *n* = 1 *n* = 1 *n* = 1 *n* = 1

BC, blood culture; GBS, group B streptococcus (*streptococcus agalactiae*); RSV, respiratory syncytial virus; respiratory infection: virus detected by multiplex PCR from nasopharyngeal aspirate. Unknown cause of fever in *n* = 14 infants (27.4%)

The control group included *n* = 40 infants < 90 days of age who were admitted to the hospital for non-infectious reasons: elective surgery for hernia or foot deformity (*n* = 25), events that required 24- to 72-hour inpatient monitoring such as apparent live-threatening events (ALTE) or check-up for seizure (*n* = 12) and hyperbilirubinemia (*n* = 3). All infants included in the control group did not show any clinical or laboratory signs of infection and were not exposed to antibiotics. Apart from higher rates of previous antibiotic treatment and reduced human milk feeding in LOS cases as compared to controls infants, there were no univariate differences between the groups (Table 1).

### Microbiological diagnostics

Urosepsis was diagnosed in 17 of 24 infants with IBI. While *E. coli* was found in 16 infants, one infant had urosepsis caused by *Klebsiella oxytoca* (*n* = 1). *Streptococcus agalactiae (GBS)* culture-proven sepsis was diagnosed in five infants, with one *GBS* meningitis and one *GBS* endocarditis case. Further causes of IBI included one blood-culture positive sepsis with *E. coli* and another blood-culture positive pneumonia with *Klebsiella pneumoniae*. Viral causes of suspected LOS included infection with enteroviruses (*n* = 6), rhinoviruses (*n* = 3), RSV (*n* = 1), coronaviruses (*n* = 2) and rotavirus (*n* = 1).

### Clinical characteristics stratified to follow-up cohort and control group

The follow-up cohort at 12 months consisted of 26 infants with suspected LOS and 21 control infants. There were no differences in gestational age, birth weight, gender, delivery mode, or any exposure to human breast milk within the first 6 and 12 months of age (Supplementary Table 1). LOS infants were older than infants in the control group at sampling (484.5 days vs. 371 days, *p* < 0.01). Importantly, LOS infants were again treated with antibiotics in 34.6% of the cases after hospital discharge within the first year of life, whereas infants from our control group received any antibiotic treatment in 9.5% of the cases within the first year. Further, infants with LOS in the neonatal period were fed with human breast milk longer (10.5 months vs. 6.0 months, *p* = 0.03), and were more often supplemented with probiotics (34.6% vs. 9.5%) within the first year of life as compared to control infants.

### Gut microbial composition at onset of sepsis differs from control infants

**Fig. 1:**
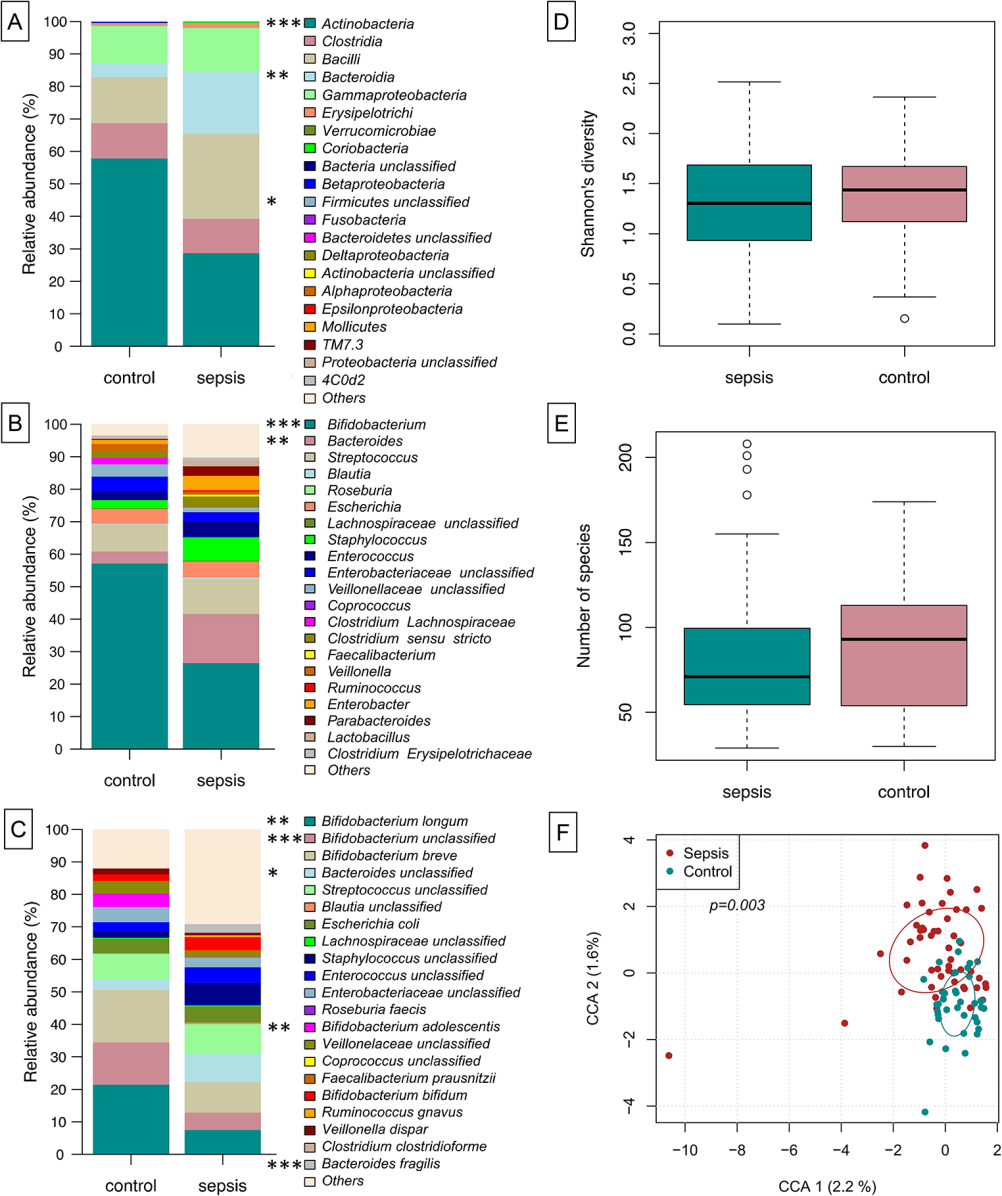
Gut microbial composition at onset of sepsis differs from healthy, same aged children. On class level, Actinobacteria are diminished in sepsis infants, while Bacteroidia are increased instead (**A**). Genus level assignment shows that the decrease in relative abundance of Actinobacteria is largely due to less of genus *Bifidobacterium*, while the genus *Bacteroides* is increased in septic children. On species level, the differences between sepsis and control group are depicted by *B. longum* and *B. adolescentis* B alongside unclassified Bifidobacteria (increased in controls) and unclassified *Bacteroides* sequences as well as *B. fragilis* (in sepsis infants) (**C**). While measures of alpha-diversity are similar between the groups (**D**-**E**), constrained correspondence analysis depicts the global changes of both groups to be significant (**F**)

**Fig. 2:**
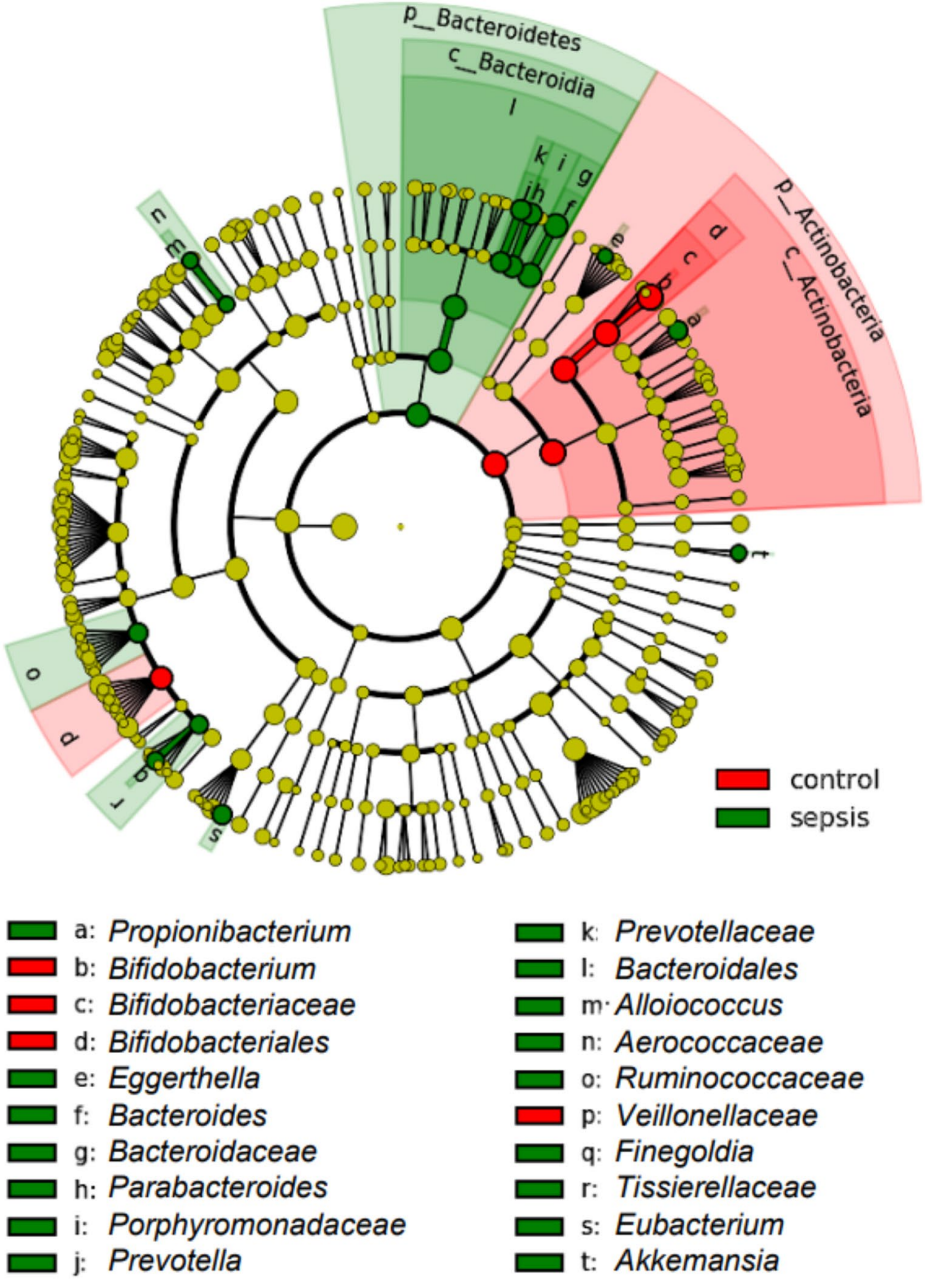
LEfSe-analysis supports the notion of the genera *Bifidobacterium* and *Bacteroides* defining hallmarks of control vs. sepsis children. Significant associations of the genus *Bifidobacterium* with the control group and *Bacteroides* with the LOS group and their respective upper taxonomic ranks is shown by linear discriminant effect size analysis (LEfSe). Additional taxa are assigned in this analysis to both of the groups

We were interested in understanding the microbial shifts occurring at the onset of a LOS period. When globally assessed, we identified in the relative abundance levels of Actinobacteria (decreased at onset of LOS, np-ANCOVA: *p* < 0.0001) and Bacteroidia (increased at LOS onset, np-ANCOVA: *p* = 0014) (Fig. 1A). In neonates, the main genus contributing to high Actinobacteria abundance was *Bifidobacterium* (np-ANCOVA: *p* = 0.0001). Accordingly, *B. longum*, *B. adolescentis* and further (unclassified) *Bifidobacterium* sequences appeared to be more prominent in control infants compared to infants with suspected LOS (np-ANCOVA: *p* = 0.0071, *p* = 0.0012 and *p* = 0.0001, respectively). The increase of Bacteroidia in the LOS cases could largely be explained by higher relative abundances of the genus *Bacteroides* (Fig. 1B, np-ANCOVA: *p* = 0.0038) and therein by *B. fragilis* and unclassified *Bacteroides* sequences (Fig. 1C, np-ANCOVA: *p* = 0.0002 and *p* = 0.049, respectively). While data comparing abundances were supported by LEfSe analysis for the genera *Bifidobacterium* and *Bacteroides*, further genera were associated with LOS: *Propionibacterium*, *Prevotella*, *Alloiococcus*, *Finegoldia* and *Akkermansia* (Fig. 2 and Suppl. Figure 2). While measures of alpha-diversity were similar between LOS and control infants (Fig. 1D-E), constrained correspondence analysis depicted the global changes of both groups to be significant (Fig. 1F, Permutation test for constrained correspondence analysis, *p* = 0.0030).

### Sustained deviation in microbial composition at one year of age

**Fig. 3:**
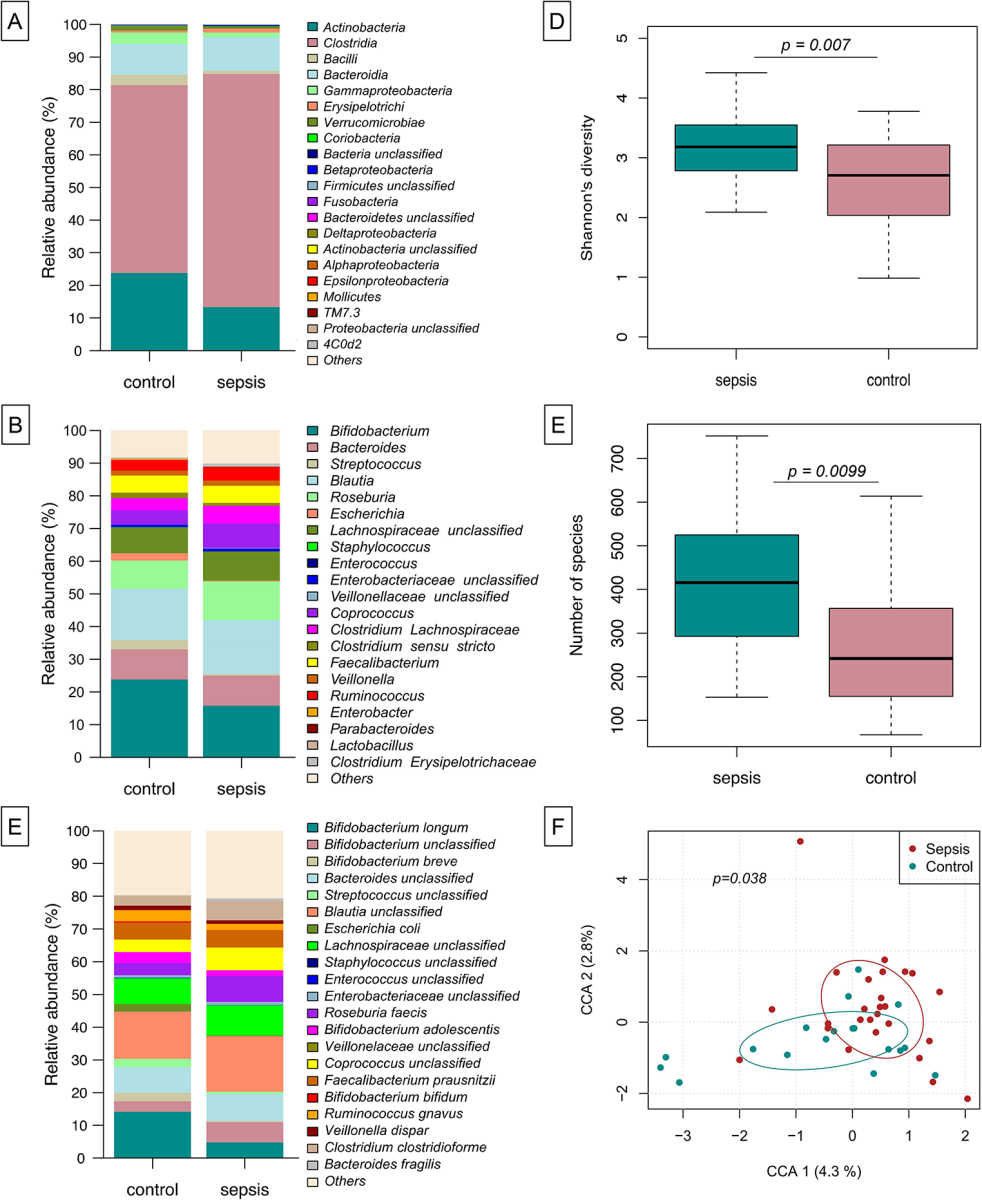
Sustained deviation in microbial composition following treatment for LOS. At age of 12 months infants’ microbiota has developed to a largely Clostridia-dominated community (**A**) with typical genera/species expected in the gut (**B**-**C**). Assessment of Shannon´s diversity index (**D**) and species richness (**E**) as well as constrained correspondence analysis (**F**) show significant differences between sepsis and controls

We were interested in whether early-in-life differences as described above may translate into long lasting developmental differences in gut colonization. We herein analyzed the gut microbiota of the infants from this cohort at 12 months of age. Both, infants with LOS in the first three months of life and control infants developed a largely Clostridia-dominated gut microbial community (Fig. 3A) with typical gut bacteria being displayed by genus and species level assignment (Fig. 3B-C). Even after this period, differences in the microbial composition were depicted regarding alpha diversity (Shannon´s diversity index and species richness, np-ANCOVA: *p* = 0.0070 and *p* = 0.0099, respectively), which was lower in controls compared to infants with early LOS) (Fig. D-E) and in constrained correspondence analysis (Fig. 3F, Permutation test for constrained correspondence analysis, *p* = 0.0380).

### Immune cell counts within the first three months of life correlate with relative abundance of the genus *Bifidobacterium* and the species *Bifidobacterium longum*

**Fig. 4:**
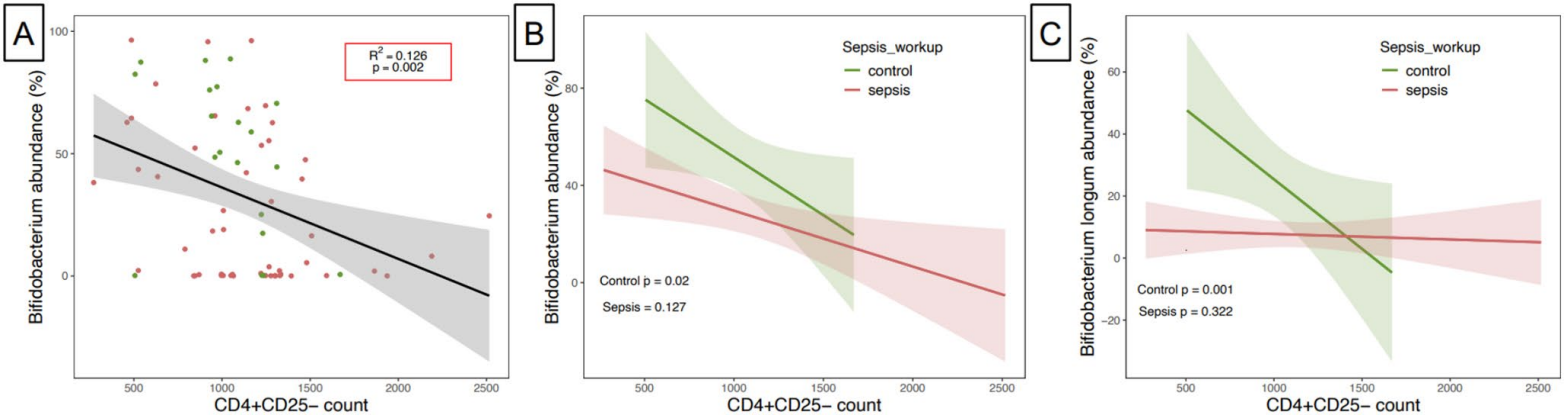
Counts of CD4 + CD25- immune cells at the age of < 90 days correlate with relative abundance of *Bifidobacterium* genus and specifically *B. longum* in multiple linear regression analysis. Genus *Bifidobacterium* is significantly negatively correlated with the abundance of CD4 + CD25- cells (**A**). However, when stratifying the analysis by LOS status the negative correlation remains significant only for the control group (**B**). This deviation between the groups is even more pronounced for the species level assignment of *B. longum*, where the negative correlation is present for the control group but completely missing for the sepsis group

We analyzed the abundance of immune cell markers in correlation to the genus *Bifidobacterium* and the most prominent bacterial species therein (*B. longum*) at LOS onset at age < 90 days. Remarkably, we noted a negative correlation between *Bifidobacterium* genus and *B. longum* relative abundance with CD4-positive/CD25-negative cells (Fig. 4A and Suppl. Figure 1) in healthy controls (Fig. 4B-C, multiple linear regression: *p* = 0.02 and *p* = 0.001, respectively), whereas no significant association for the genus *Bifidobacterium* (Fig. 4B) was found in the sepsis cases. The low relative abundance of *B. longum* in LOS cases had no apparent effect on CD4 positive cells (Fig. C). High values of CD4-positive/CD25-negative cells were only demonstrated in cases characterized by a low relative abundance of bifidobacteria (Figs. 1B and 4A). The spread of abundance values of immune cells was higher in the LOS group for cells which correlated to microbial factors in this study. Unlike the above-mentioned CD4positive/CD25-negative cells, other tested immune cells were not associated with changes in both genus *Bifidobacterium* and *B. longum* abundance (Suppl. Figure 1).

## Discussion

In this prospective single-center case-control study we present unique microbiome-immunity data of young febrile infants < 90 days of age with suspected community-acquired LOS. At onset of infection infants were characterized by significant changes of the gut microbiome, such as a reduced abundance of *Bifidobacterium* spp. and an increase in *Bacteroides* spp. as compared to unaffected controls. At 12-month-follow-up, infants showed different microbiome characteristics when compared to healthy controls, depicted by increased gut microbial diversity and deviation from the control group in constrained correspondence analysis.

Our explorative study generates the hypothesis that microbiome immaturity traits have a reciprocal influence on the immune system and are specifically associated with the onset of LOS. Our observation of a reduced abundance of *Bifidobacterium* spp. in LOS infants is supported by previous studies of preterm infants displaying a lack of *Bifidobacterium* spp./ Actinobacteria before the onset of LOS [11]. Specifically, *B. longum* is a main driver of the neonatal intestinal microbiome and metabolome and plays an important role by competing with potential pathogenic bacteria such as *Staphylococcus* spp., *Klebsiella* spp. and *Enterobacteriaceae* [39]. Our data postulates a key role of *B. longum* in interaction with certain T cell subsets. Specifically, we noted that high abundance of *B. longum* correlates with low CD4^+^ /CD25^−^ cells in healthy controls, while this correlation is absent in infants with LOS. Second, gut colonization with anaerobic bacteria, such as *Bifidobacterium* spp., is known to be important in preventing translocation sepsis [40, 41] by stabilizing epithelial gut integrity and enhancing barrier function [42]. Hence, a lack of bifidobacterial colonization could be critical for infection risk during the postnatal shift from aerobic to anaerobic microbiota species [43]. Further, the infants’ gut colonization with *Bifidobacterium* spp. is known to be associated with beneficial development of postnatal immune tolerance [44, 45]. The reciprocal correlation between *B. longum* and CD4 + lymphocytes in controls was abolished in our target population. CD4 + lymphocytes are known to be influenced by the microbiome in terms of function and polarization in different ways [23] and their metabolism is influenced by microbes during inflammation [23]. It has recently been shown, that *B. longum* subsp. *infantis* is able to mitigate Th1 polarization that would otherwise be induced by different *Staphylococcus* species [46]. T-helper cells react differently to infections depending on the microbes present in the infant gut. In this light, our observation supports the hypothesis of disturbed bilateral microbiome-immunity interaction in infants with LOS, where key players of the establishing microbiome such as *B. longum* form a robust immunological function and, conversely, the immune system enables successful integration of microbiota into the microbiome.

It is important to recognize that our subgroups differed in baseline characteristics such as exposure to breast milk and mode of birth, both of which significantly impact the microbiome composition in the first weeks of life. Breast milk, as a source of *Bifidobacterium*, *Lactobacillus*, *Enterococcus*, and *Staphylococcus* species, plays a crucial role in shaping the infant gut microbiome [47]. Infants born vaginally are initially colonized by microbes from the maternal rectovaginal flora, predominantly *Lactobacillus*, *Prevotella*, or *Sneathia* species. In contrast, the early microbiome of infants delivered via caesarean section is dominated by skin bacteria such as *Staphylococcus*, *Corynebacterium*, and *Propionibacterium* species [48]. We conducted our analyses while considering these characteristics as confounders and adjusted our statistical analysis accordingly. However, due to our explorative study design it remains speculative whether the microbiome differences observed between the groups are attributable to these major confounding factors or whether they are the cause or consequence of LOS. Given that LOS infants are characterized by lower abundances of bifidobacteria, we may hypothesize, that they lack the crucial sources of these bacteria, specifically contact with the birth canal during delivery and breast milk.

Known reasons for delayed microbiota maturation are preterm birth, formula feeding, low intake of breast milk, antibiotic exposure and cesarean sections [49–51]. In line with that, all infants in this vulnerable cohort are treated with broadspectrum antibiotics, usually with a triple combination of ampicillin, gentamicin and cefotaxime for a median of 7 days. Disturbances in this critical period of early microbiome development (infants < 90 days) has been hypothesized to cause disruption of healthy host-commensal interactions leading to persistent and potentially irreversible defects in the development and training of specific immune subsets [52–54]. In our cohort, the increased diversity in infants with LOS and consecutive early antibiotic exposure may point towards an unstable gut microbial composition, that is usually largely dominated by *Clostridium* spp. in healthy one-year old children. However, we must mention that we did not identify differences at the genus or species level, which largely hampers assumptions about clinical relevance in the long-term such as asthma, obesity, diabetes and inflammatory diseases [55, 56]. In addition, most microbiome studies investigating the aforementioned adverse long-term outcomes have demonstrated associations with reduced, rather than increased, microbial diversity [57]. These uncertainties must be addressed in future research in order to establish antibiotic stewardship programs that effectively protect infants from long-term consequences of antibiotic exposure. Of note, the high rate of probiotic supplementation after hospital discharge in infants with antibiotic exposure due to LOS treatment (42.3%) may reflect post treatment symptoms leading families to seek options to beneficially modulate the microbiome. The evidence for probiotics after antibiotic treatment in infancy is lacking [58, 59].

### Strengths and limitations

The major strength of our study is the unique cohort of previously healthy infants < 90 days of age with suspected LOS. There are potential limitations to our study, i.e. the single center approach. All samples were collected from symptomatic infants, so it cannot be proven whether our findings are the cause or the consequence of infection. Our study describes quantitative data, whereas functional capacities or single cell signatures are not yet studied in detail. In order to study immunological characteristics that predispose young infants to sepsis, longitudinal analyses are needed that require larger cohorts of term infants at risk for LOS. The limited sample size of our cohort restricts the validity of a subgroup analysis that takes into account the cause of fever (viral vs. bacterial). Due to the study design and the unpredictable nature of sepsis presentation, it was not feasible to process samples in balanced batches of LOS infants and controls, which represents a possible source of bias. However, we ensure that all samples were processed using the same standardized protocols to maintain consistency and reliability of our analyses. For the 12-months-follow-up observations, it remains speculative whether the long-term gut microbial deviations result from infection, early antibiotic exposure, diet or a combined predisposition of the microbiome – immunity interplay with a yet unknown effect on long-term health. Finally, this exploratory study can only partially compensate for the numerous confounding factors of the microbiome composition in the first year of life, such as diet, antibiotic and probiotic exposure. However, hypotheses within this important field of research (antibiotic stewardship) warrant further investigation in future studies to identify possible prevention targets.

## Electronic supplementary material

Below is the link to the electronic supplementary material.

Supplementary Material 1

## Data Availability

The raw sequencing data are freely available online at the European Nucleotide Archive under accession number [PRJEB68362].

## Abbreviations

FWS: Fever without source
GBS: Group-B-Streptococcus (*Streptococcus agalactiae*)
IBI: Invasive bacterial infection
LOS: Late onset sepsis
Treg: Regulatory T cell
CCA: Constraint correspondence analysis
np-ANCOVA: Non-parametric analysis of covariances
