# Supplementary material for: Infants < 90 days of age with late-onset sepsis display disturbances of the microbiome-immunity interplay
Source: Infection. 2024 Nov 14;53(3):921–34. doi: 10.1007/s15010-024-02396-6 (PMC12137456; doi:10.1007/s15010-024-02396-6)
Supplement: Supplementary file 1 — Supplementary Material 1 [file 15010_2024_2396_MOESM1_ESM.docx]

***Supplementary table 1.*** *Clinical characteristics of follow-up cohort at 12 months of age*

|  | **Antibiotic**  **Exposure (LOS)** | **Control**  **group^#^** | **p** |
| --- | --- | --- | --- |
| **Number** n | 26 | 21 | ♦ |
| **Gestational age**  (weeks; median, IQR) | 39.6  (38.2 – 40.3) | 39.0  (38.1 - 39.7) | 0.3 |
| **Birth weight**  (g; median, IQR) | 3538  (3150 – 3945) | 3480  (3120 – 3650) | 0.3 |
| **Gender, male** (%, n) | 61.5  (n=16) | 61.9  (n=13) | 0.9 |
| **Delivery mode, vaginal delivery** (%, n) | 76.9  (n=20) | 57.1  (n=12) | 0.1 |
| **Age at follow-up**  (days; median, IQR) | 484.5  (400 – 574.3) | 371  (361.5 – 397.5) | < 0.01 |
| **Antibiotic exposure within first year***  (%, n) | 34.6  (n=9) | 9.5  (n=2) | 0.04 |
| **Human milk-fed (any time)**  (%, n) | 84.0  (n =21) | 65.0  (n =13) | 0.1 |
| **HM-fed for at least 6 months**  (%, n) | 61.5  (n=16) | 52.4  (n=11) | 0.5 |
| **HM-fed until 12 months**  (%, n) | 46.2  (n=12) | 25.0  (n=5) | 0.1 |
| **Duration of HM exposure**  (months; median, IQR) | 10.5  (4.0-13.0) | 6.0  (0.5-11.5) | 0.03 |
| **Formula-fed**  (%, n) | 38.4  (n =10) | 61.6  (n =13) | 0.1 |
| **Probiotic supplementation within first year** (%, n) | 42.3  (n=11) | 9.5  (n=2) | 0.04 |

***Legend:*** *IBI, invasive bacterial infection; IQR interquartile range;* ***#*** *p-value were derived from Chi-Square test or for continuous variables Mann-Whitney U test.*

* *No relation LOS treatment.*

^♦^ *^Exclusion criteria for controls or not applicable.^*

**Supplementary Table 1: List of primers used for amplification of the V3/V4 hypervariable region of microbial 16S rRNA gene for next generation sequencing**

| Oligo name | Sequence 5’-3’ | Index |
| --- | --- | --- |
| hV3F_MID1 | AATGATACGGCGACCACCGAGATCTACAC ATCGTACG ACACTCTTTCCCTACACGACGCTCTTCCGATCT CCTACGGGAGGCAGCAG | ATCGTACG |
| hV3F_MID2 | AATGATACGGCGACCACCGAGATCTACAC ACTATCTG ACACTCTTTCCCTACACGACGCTCTTCCGATCT T CCTACGGGAGGCAGCAG | ACTATCTG |
| hV3F_MID3 | AATGATACGGCGACCACCGAGATCTACAC TAGCGAGT ACACTCTTTCCCTACACGACGCTCTTCCGATCT GT CCTACGGGAGGCAGCAG | TAGCGAGT |
| hV3F_MID4 | AATGATACGGCGACCACCGAGATCTACAC CTGCGTGT ACACTCTTTCCCTACACGACGCTCTTCCGATCT CGA CCTACGGGAGGCAGCAG | CTGCGTGT |
| hV3F_MID5 | AATGATACGGCGACCACCGAGATCTACAC TCATCGAG ACACTCTTTCCCTACACGACGCTCTTCCGATCT ATGA CCTACGGGAGGCAGCAG | TCATCGAG |
| hV3F_MID6 | AATGATACGGCGACCACCGAGATCTACAC CGTGAGTG ACACTCTTTCCCTACACGACGCTCTTCCGATCT TGCGA CCTACGGGAGGCAGCAG | CGTGAGTG |
| hV3F_MID7 | AATGATACGGCGACCACCGAGATCTACAC GGATATCT ACACTCTTTCCCTACACGACGCTCTTCCGATCT GAGTGG CCTACGGGAGGCAGCAG | GGATATCT |
| hV3F_MID8 | AATGATACGGCGACCACCGAGATCTACAC GACACCGT ACACTCTTTCCCTACACGACGCTCTTCCGATCT CCTACGGGAGGCAGCAG | GACACCGT |
| hV3F_MID9 | AATGATACGGCGACCACCGAGATCTACAC CTACTATA ACACTCTTTCCCTACACGACGCTCTTCCGATCT T CCTACGGGAGGCAGCAG | CTACTATA |
| hV3F_MID10 | AATGATACGGCGACCACCGAGATCTACAC CGTTACTA ACACTCTTTCCCTACACGACGCTCTTCCGATCT GT CCTACGGGAGGCAGCAG | CGTTACTA |
| hV3F_MID11 | AATGATACGGCGACCACCGAGATCTACAC AGAGTCAC ACACTCTTTCCCTACACGACGCTCTTCCGATCT CGA CCTACGGGAGGCAGCAG | AGAGTCAC |
| hV3F_MID12 | AATGATACGGCGACCACCGAGATCTACAC TACGAGAC ACACTCTTTCCCTACACGACGCTCTTCCGATCT ATGA CCTACGGGAGGCAGCAG | TACGAGAC |
| hV3F_MID13 | AATGATACGGCGACCACCGAGATCTACAC ACGTCTCG ACACTCTTTCCCTACACGACGCTCTTCCGATCT TGCGA CCTACGGGAGGCAGCAG | ACGTCTCG |
| hV3F_MID14 | AATGATACGGCGACCACCGAGATCTACAC TCGACGAG ACACTCTTTCCCTACACGACGCTCTTCCGATCT GAGTGG CCTACGGGAGGCAGCAG | TCGACGAG |
| hV3F_MID15 | AATGATACGGCGACCACCGAGATCTACAC GATCGTGT ACACTCTTTCCCTACACGACGCTCTTCCGATCT CCTACGGGAGGCAGCAG | GATCGTGT |
| hV3F_MID16 | AATGATACGGCGACCACCGAGATCTACAC GTCAGATA ACACTCTTTCCCTACACGACGCTCTTCCGATCT T CCTACGGGAGGCAGCAG | GTCAGATA |
| hV3F_MID17 | AATGATACGGCGACCACCGAGATCTACAC ACGACGTG ACACTCTTTCCCTACACGACGCTCTTCCGATCT GT CCTACGGGAGGCAGCAG | ACGACGTG |
| hV3F_MID18 | AATGATACGGCGACCACCGAGATCTACAC CGTCGCTA ACACTCTTTCCCTACACGACGCTCTTCCGATCT CGA CCTACGGGAGGCAGCAG | CGTCGCTA |
| hV3F_MID19 | AATGATACGGCGACCACCGAGATCTACAC GCTCTAGT ACACTCTTTCCCTACACGACGCTCTTCCGATCT ATGA CCTACGGGAGGCAGCAG | GCTCTAGT |
| hV3F_MID20 | AATGATACGGCGACCACCGAGATCTACAC TGCGTACG ACACTCTTTCCCTACACGACGCTCTTCCGATCT TGCGA CCTACGGGAGGCAGCAG | TGCGTACG |
| hV4R_MID_A | CAAGCAGAAGACGGCATACGAGAT AACTCTCG GTGACTGGAGTTCAGACGTGTGCTCTTCCGATCT GGACTACHVGGGTWTCTAAT | CGAGAGTT |
| hV4R_MID_B | CAAGCAGAAGACGGCATACGAGAT ACTATGTC GTGACTGGAGTTCAGACGTGTGCTCTTCCGATCT A GGACTACHVGGGTWTCTAAT | GACATAGT |
| hV4R_MID_C | CAAGCAGAAGACGGCATACGAGAT AGTAGCGT GTGACTGGAGTTCAGACGTGTGCTCTTCCGATCT TC GGACTACHVGGGTWTCTAAT | ACGCTACT |
| hV4R_MID_D | CAAGCAGAAGACGGCATACGAGAT CAGTGAGT GTGACTGGAGTTCAGACGTGTGCTCTTCCGATCT CTA GGACTACHVGGGTWTCTAAT | ACTCACTG |
| hV4R_MID_E | CAAGCAGAAGACGGCATACGAGAT CGTACTCA GTGACTGGAGTTCAGACGTGTGCTCTTCCGATCT GATA GGACTACHVGGGTWTCTAAT | TGAGTACG |
| hV4R_MID_F | CAAGCAGAAGACGGCATACGAGAT CTACGCAG GTGACTGGAGTTCAGACGTGTGCTCTTCCGATCT ACTCA GGACTACHVGGGTWTCTAAT | CTGCGTAG |
| hV4R_MID_G | CAAGCAGAAGACGGCATACGAGAT GGAGACTA GTGACTGGAGTTCAGACGTGTGCTCTTCCGATCT TTCTCT GGACTACHVGGGTWTCTAAT | TAGTCTCC |
| hV4R_MID_H | CAAGCAGAAGACGGCATACGAGAT GTCGCTCG GTGACTGGAGTTCAGACGTGTGCTCTTCCGATCT GGACTACHVGGGTWTCTAAT | CGAGCGAC |
| hV4R_MID_I | CAAGCAGAAGACGGCATACGAGAT GTCGTAGT GTGACTGGAGTTCAGACGTGTGCTCTTCCGATCT A GGACTACHVGGGTWTCTAAT | ACTACGAC |
| hV4R_MID_J | CAAGCAGAAGACGGCATACGAGAT TAGCAGAC GTGACTGGAGTTCAGACGTGTGCTCTTCCGATCT TC GGACTACHVGGGTWTCTAAT | GTCTGCTA |
| hV4R_MID_K | CAAGCAGAAGACGGCATACGAGAT TCATAGAC GTGACTGGAGTTCAGACGTGTGCTCTTCCGATCT CTA GGACTACHVGGGTWTCTAAT | GTCTATGA |
| hV4R_MID_L | CAAGCAGAAGACGGCATACGAGAT TCGCTATA GTGACTGGAGTTCAGACGTGTGCTCTTCCGATCT GATA GGACTACHVGGGTWTCTAAT | TATAGCGA |
| hV4R_MID_M | CAAGCAGAAGACGGCATACGAGAT AAGTCGAG GTGACTGGAGTTCAGACGTGTGCTCTTCCGATCT ACTCA GGACTACHVGGGTWTCTAAT | CTCGACTT |
| hV4R_MID_N | CAAGCAGAAGACGGCATACGAGAT ATACTTCG GTGACTGGAGTTCAGACGTGTGCTCTTCCGATCT TTCTCT GGACTACHVGGGTWTCTAAT | CGAAGTAT |
| hV4R_MID_O | CAAGCAGAAGACGGCATACGAGAT CATAGAGA GTGACTGGAGTTCAGACGTGTGCTCTTCCGATCT GGACTACHVGGGTWTCTAAT | TCTCTATG |
| hV4R_MID_P | CAAGCAGAAGACGGCATACGAGAT CGTAGATC GTGACTGGAGTTCAGACGTGTGCTCTTCCGATCT A GGACTACHVGGGTWTCTAAT | GATCTACG |
| hV4R_MID_Q | CAAGCAGAAGACGGCATACGAGAT GCGCACGT GTGACTGGAGTTCAGACGTGTGCTCTTCCGATCT TC GGACTACHVGGGTWTCTAAT | ACGTGCGC |
| hV4R_MID_R | CAAGCAGAAGACGGCATACGAGAT GGTACTAT GTGACTGGAGTTCAGACGTGTGCTCTTCCGATCT CTA GGACTACHVGGGTWTCTAAT | ATAGTACC |
| hV4R_MID_S | CAAGCAGAAGACGGCATACGAGAT TACGAGCA GTGACTGGAGTTCAGACGTGTGCTCTTCCGATCT GATA GGACTACHVGGGTWTCTAAT | TGCTCGTA |
| hV4R_MID_T | CAAGCAGAAGACGGCATACGAGAT TCAGCGTT GTGACTGGAGTTCAGACGTGTGCTCTTCCGATCT ACTCA GGACTACHVGGGTWTCTAAT | AACGCTGA |

**
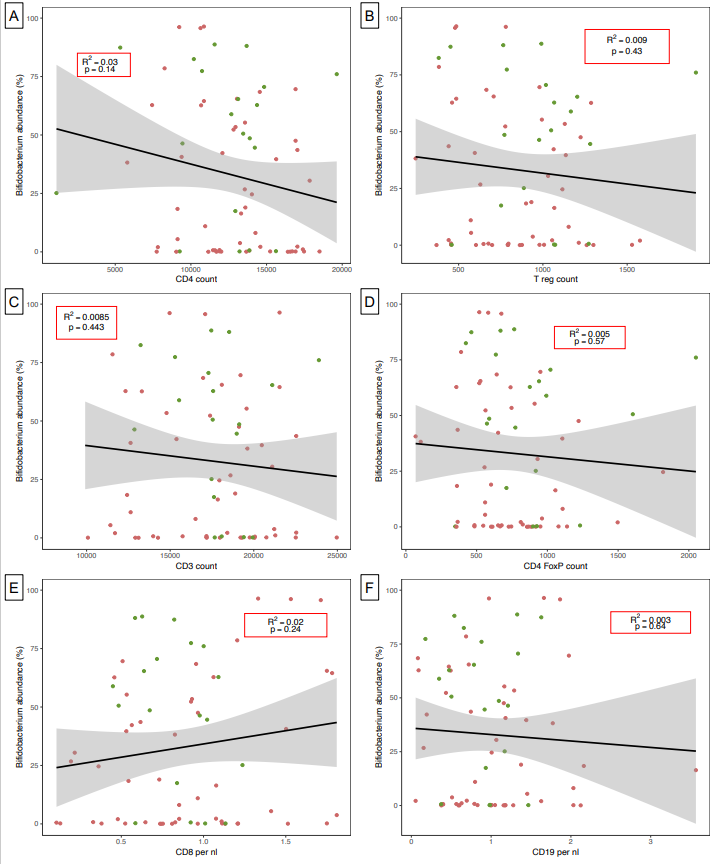
**

***Supplementary Figure 1.*** *Correlation analysis between relative abundance of the genus Bifodobacteria and immune marker counts at time of LOS including matched controls. R² and p-values from multiple linear regression models are given in each panel of the figure. Correlations with a p-value <0.05 are depicted including stratification based on LOS/control grouping in Figure 3 within the main text.*


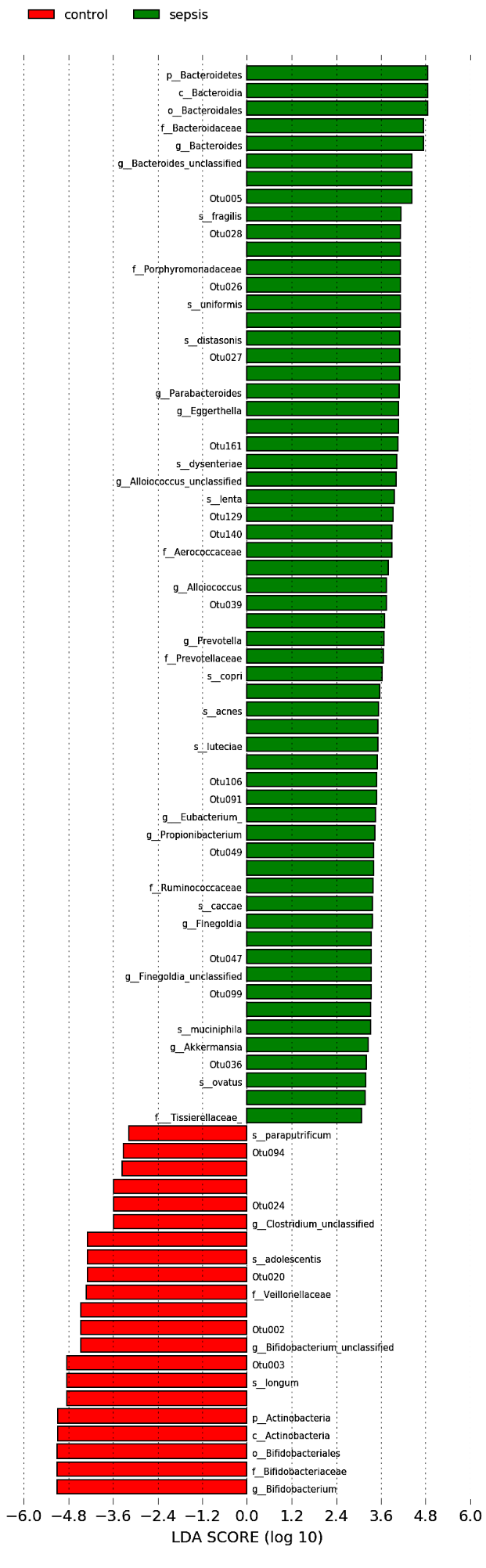


***Supplementary Figure 2.*** *Detailed depiction of LDA scores for LEFSE analysis depicted in Figure 1C within the main text.*


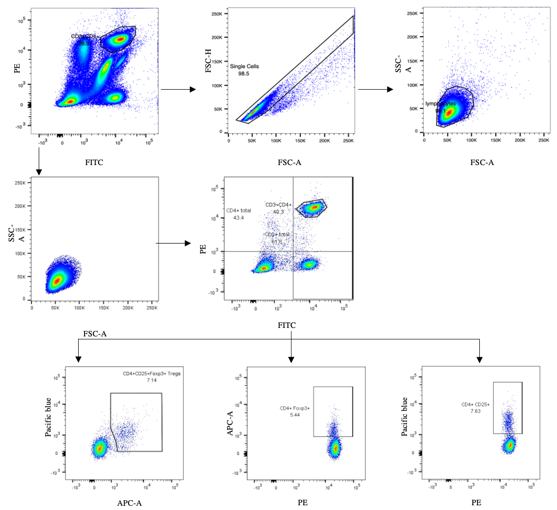


***Suppl. Figure 3.*** *Representative plots for the analyses of different lymphocyte subsets via flow cytometry and schematic representation of the applied gating strategy for the flow cytometric analysis of CD3+, CD4+, CD25+, forkhead box protein 3 (FoxP3+) regulatory T cells. [9, 15]. The gating strategy is based on the fact that many events were registered through flow cytometry (>100.000 events per sample). CD3+ CD4+ cells were gated before the lymphocytes in order to more easily identify lymphocytes. CD3+ CD4+ cells were gated, a single cell gate was set to exclude doublets and the lymphocytes were determined in the initial steps. Afterwards the lymphocyte gate was backgated on the first population and calculated to a total number of 30.0000 cells in the backgated lymphocyte gate. Total CD3+, CD4+ and CD3+CD4+ cells were determined. These were further analyzed for the gating of the following subpopulations: CD3+CD4+Foxp3+ Tregs, CD3+CD4+Foxp3+ and CD3+CD4+CD25+ cells. Absolute cell counts and frequencies of cells were calculated, as well as the mean fluorescence intensity of the APC-A channel, which detects the Foxp3 monoclonal antibody with eFlour660 fluorochrome. The numbers in the panels indicate the frequency of the gated cells in percentages. FSC, forward-scatter; SSC, side-scatter.*
